# Deep Brain Stimulation Programming for Movement Disorders: Current Concepts and Evidence-Based Strategies

**DOI:** 10.3389/fneur.2019.00410

**Published:** 2019-05-21

**Authors:** Thomas Koeglsperger, Carla Palleis, Franz Hell, Jan H. Mehrkens, Kai Bötzel

**Affiliations:** ^1^Department of Neurology, Ludwig Maximilians University, Munich, Germany; ^2^Department of Translational Neurodegeneration, German Center for Neurodegenerative Diseases (DZNE), Munich, Germany; ^3^Graduate School of Systemic Neurosciences, Ludwig-Maximilians-Universität München, Martinsried, Germany; ^4^Department of Neurosurgery, Ludwig Maximilians University, Munich, Germany

**Keywords:** DBS programming algorithms, subthalamic nucleus, DBS side effects, segmented electrode, short pulse width

## Abstract

Deep brain stimulation (DBS) has become the treatment of choice for advanced stages of Parkinson's disease, medically intractable essential tremor, and complicated segmental and generalized dystonia. In addition to accurate electrode placement in the target area, effective programming of DBS devices is considered the most important factor for the individual outcome after DBS. Programming of the implanted pulse generator (IPG) is the only modifiable factor once DBS leads have been implanted and it becomes even more relevant in cases in which the electrodes are located at the border of the intended target structure and when side effects become challenging. At present, adjusting stimulation parameters depends to a large extent on personal experience. Based on a comprehensive literature search, we here summarize previous studies that examined the significance of distinct stimulation strategies for ameliorating disease signs and symptoms. We assess the effect of adjusting the stimulus amplitude (A), frequency (f), and pulse width (pw) on clinical symptoms and examine more recent techniques for modulating neuronal elements by electrical stimulation, such as interleaving (Medtronic®) or directional current steering (Boston Scientific®, Abbott®). We thus provide an evidence-based strategy for achieving the best clinical effect with different disorders and avoiding adverse effects in DBS of the subthalamic nucleus (STN), the ventro-intermedius nucleus (VIM), and the globus pallidus internus (GPi).

## Introduction

Since the pioneering work of Cooper et al. (1) and of Benabid et al. in the early 1990s (2), deep brain stimulation (DBS) has become the treatment of choice for advanced stages of Parkinson's disease (PD), for medically intractable essential tremor (ET), and for complicated segmental and generalized dystonia. Although overall considered an effective treatment in these diseases, a number of specific factors determine the treatment success: in addition to careful patient selection and accurate electrode placement, the effective post-operative programming of DBS devices is considered the most important factor for the individual patient outcome (3–5). Programming is the only modifiable factor once a patient has been implanted with DBS leads and it becomes even more relevant in cases in which the DBS electrodes are located at the border of the intended target structure. Current implantation techniques, using either stereotaxic frames or surgical robots, exhibit an average precision in the range of 1–2 mm from the target area (6–12). In addition, the brain itself can shift by 2–4 mm during surgery (13–15), contributing to imprecise lead placement. According to previous studies, such errors occur in up to 40% of DBS surgeries (16–20), thus underscoring the importance of post-operative programming to compensate for such variability. Inefficient stimulation may result in unnecessary follow-up visits and reduced patient satisfaction with DBS (21). Conversely, sound programming has been shown to improve patient outcomes and to avoid unnecessary lead revisions (19). In addition, improvement with re-programming highlights that proper adjustment of stimulation parameters is a major factor for successful treatment and patient satisfaction (22).

**Figure 1:**
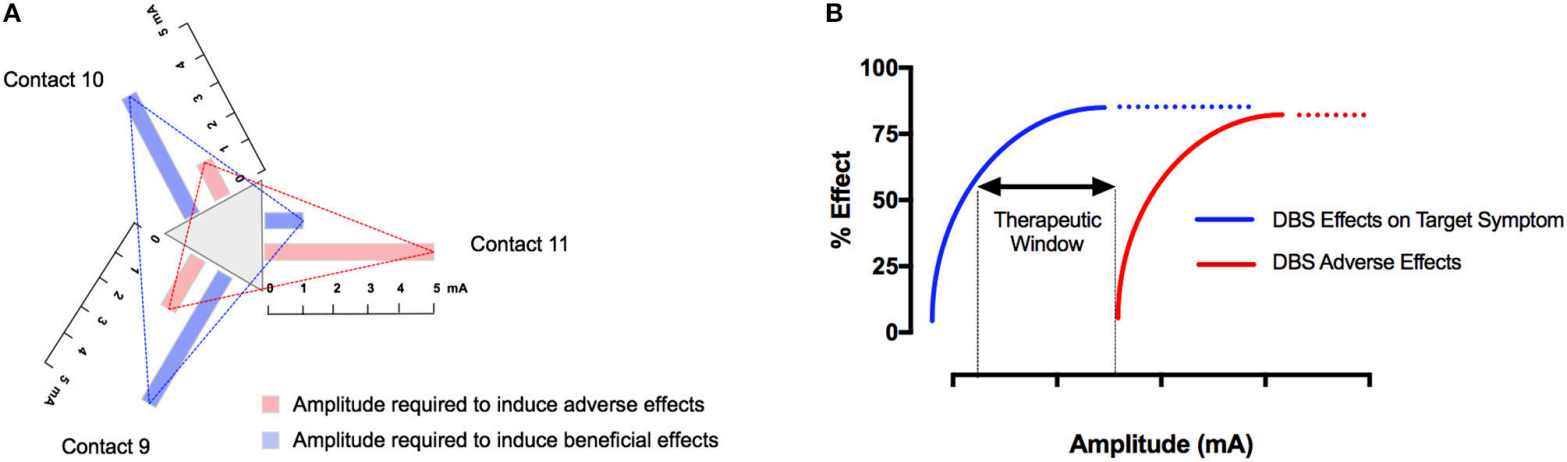
The therapeutic window depends on stimulation parameters and the electrode configuration. In tripartite electrodes, the therapeutic window should be determined for each segment individually by examining the beneficial and adverse effects with increasing the stimulation amplitude under defined pulse width and frequency **(A)**. The therapeutic window in DBS is defined as the gap between the minimum stimulation current required to produce adverse effects and the current required to produce a beneficial effect. Similar to pharmacologic intervention, DSB is a tradeoff between beneficial and adverse effects. Numerous stimulation parameters, as well as the anatomical position of the respective contact, affect the therapeutic window. As a consequence, each electrode contact and each combination of pulse width and frequency thus has an individual therapeutic window **(B)**.

Despite established strategies for adjusting neurostimulation (23–27), DBS programming remains time- and resource-consuming. New leads with two levels of tripartite electrodes (i.e., segmented electrodes) (Abbott®, Boston Scientific®) can improve the therapeutic window (Figures 1A,B) but increase the number of possible combinations of programming parameters (28) [For a thorough review of currently implanted pulse generators (IPGs) and electrodes see: (29)]. Therefore, there is a need for sophisticated strategies on how to adjust stimulation parameters and lead configurations in a precise and effective manner once the electrodes have been implanted. We here review the current evidence for adjusting neurostimulation in different movement disorders. Regarding the biophysical and physiological effects of DBS, the reader is referred to extensive reviews on this matter (30, 31).

## Current Programming Strategies

### Specific Programming Strategies for DBS of the Subthalamic Nucleus (STN)

It is thought that adjustment of stimulation parameters is best carried out by trained clinicians (3) and depends to a large extent on personal experience, whereas detailed algorithms for a disease-specific programming strategy are rare, with the exception of expert recommendations (3, 27, 32).

#### Assessing the Response to DBS:

**Figure 2:**
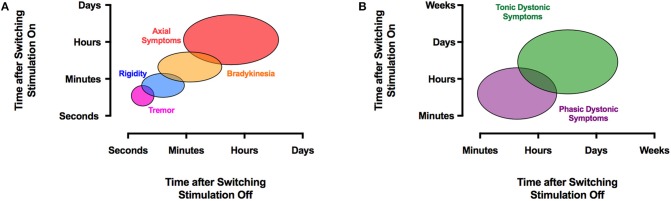
The effects of DBS on clinical symptoms are time-dependent. PD signs and symptoms respond to STN-DBS variably. Axial symptoms may take hours or days to improve, whereas tremor typically disappears almost instantly with STN- or VIM-DBS **(A)**. A similar temporal disparity occurs with dystonia, where phasic dystonic symptoms respond quickly within minutes to GPi-DBS, and tonic dystonic movements may take much longer to resolve **(B)**. The reappearance of symptoms after discontinuation of DBS exhibits a similar temporal pattern.

In order to judge the effect of STN-DBS, rigidity is typically used in PD because it does not fluctuate, responds to stimulation adjustments within seconds (Figure 2A), and does not depend on the patient's fatigue or cooperation (33, 34). When effective stimulation is switched on, rigidity disappears within 20 s, whereas after cessation of stimulation, rigidity returns within 1 min (35) (Figure 2A). This must be taken into account when subsequent tests are performed. In the absence of rigidity, bradykinesia or (rest) tremor can be used, although the response of bradykinesia to changing the stimulation parameters is slower (33) and may be biased by fatigue and the patient's discomfort or expectations and (rest) tremor may fluctuate spontaneously. Gait speed, arm swing during gait, finger tapping, or alternating hand movements can all be measured with a stopwatch to achieve numeric data to supply evidence for a certain stimulator setting. A list of appropriate tests has been suggested (36). Also, selected items from the UPDRS-III scale are used to judge the therapeutic effect and to document effects in a systematic manner. It is noteworthy that no single clinical sign or symptom should be used alone (such as e.g., rigidity) to judge the therapeutic effect. Our clinical experience suggests that one should select from a list of possible tests two or three which characterize the symptoms of the patient best and to apply these tests in a systematic manner during the programming sessions. The contact with the lowest threshold for beneficial effects and the widest therapeutic window is then selected for chronic stimulation (23–27).

#### Electrode Configuration Adjustment

It is commonly suggested that once the leads have been implanted, each ring contact should be tested in a monopolar configuration with the electrode as negative (cathode) and the IPG as positive (anode), a process referred to as monopolar review (3, 27, 32). In some centers, this is done prior to the implantation of the IPG using externalized leads, with the option to adjust the depth of the implanted electrode during the implantation of the IPG. In these cases, stimulation is applied by an external stimulator. Initially, the pulse width and frequency are kept constant at 60 μs and 130 Hz, respectively. Each of the ring electrodes is tested separately with increasing amplitudes to determine the threshold of beneficial effects and, with further increasing the amplitude, to detect the threshold of adverse effects (3, 37). In the case of segmented electrodes, all segments of one ring are activated simultaneously (38). Most authors suggest a gradual increase of stimulation amplitude in steps of 0.1–0.5 V or 0.1–0.5 mA up to a maximum of 5 V or 5 mA, or until side effects occur (3, 25, 37).

When newer DBS leads (Boston Scientific®, Abbott®) with two levels of tripartite electrodes are used, it is suggested that after determination of the clinically most efficient ring, single contacts of this ring are screened in a similar fashion (directional or current steering) (39, 40) (Figures 1A,B). Stimulation of single segments can result in a larger therapeutic window (38). In addition, the average current threshold for obtaining a therapeutic effect was noted to be lower with the best directional stimulation (41–44). In accord, Pollo et al. reported, in their study on intraoperative segmental stimulation, a reduced threshold for clinical efficiency as well as a better clinical efficiency with segmental stimulation (39). Even with small currents of 0.3 mA, these authors were able to induce clinical effects in individual patients, which suggests that the stepwise increase of current during testing may have to be considerably lower than 0.5 mA. In the VANTAGE study, stimulation was performed with the Vercise system (Boston Scientific®) that includes a separate current source for each segment of the lead which contains 8 contacts (45). These authors stimulated the best as well as the second best segment and instructed their patients to optimize the applied current via a patient control device. The authors reported an improvement of over 60% during the ON phase on the UPDRS-III rating scale, which is above the average improvement seen with conventional ring electrodes.

#### Stimulation Parameter Selection

In order to achieve the best clinical effect, certain stimulation parameters have been determined empirically for STN-DBS. Previous studies investigating the specific contribution of frequency, pulse width, and amplitude found that the amplitude had the greatest effect on ameliorating PD motor signs relative to energy-equivalent changes in frequency and pulse width (23, 24). In one study that examined PD patients with STN-DBS, the amplitude required to improve wrist rigidity ranged from 0.7 to 1.7 mA, and the amplitude required to generate adverse effects was in the range of 1.3–3.4 mA (23). In an intraoperative examination of clinical STN-DBS effects in 17 PD patients, Sauleau et al. found that the threshold for the vanishing of wrist rigidity was 0.94 V (at 130 Hz and 100 μs) (46). Stimulation frequencies of 50 Hz and 130 Hz improved tremor, rigidity, and bradykinesia, with rigidity improving already above a threshold of 33 Hz. In these studies, there was no significant improvement above 185 Hz for either target symptom, although some reports suggest that tremor tends to respond to a higher frequency (47). Using frequencies below 50 Hz in STN-DBS did not improve motor signs, even when the total electrical energy delivered (TEED) was similar (23). In fact, very low frequencies of 5–10 Hz have been found to worsen motor symptoms, in particular, bradykinesia, compared with no stimulation (24, 48, 49). Moro et al. demonstrated that pulse widths between 60 and 210 μs were beneficial for improving tremor and rigidity, while reduction of bradykinesia relative to baseline was only significant at 60 μs. High-pulse-width stimulation (>210 μs) was generally not well-tolerated. No difference in tremor has been observed with different pulse widths (23, 24). In addition to rigidity, tremor, and akinesia, STN-DB has a beneficial effect on off-dystonia (50, 51), whereas improvement in on-dyskinesia is predominantly a consequence of a reduced L-Dopa equivalent dose (LED) (52). Recently, IPGs became available which allow for even shorter pulse widths 60 μs. The CUSTOM-DBS study by Steigerwald et al. investigated 15 PD patients with STN-DBS and found that for STN stimulation, a shorter pulse width of 30 μs resulted in a larger therapeutic window with a non-inferior therapeutic efficacy (as measured by the UPDRS III score) when compared to the standard pulse width of 60 μs (53). Also, another group showed that stimulation using 30 μs pulse-width results in better walking and speech performance at a similar total electrical energy delivered (TEED) (54). Therefore, the previous recommendation for a fixed pulse width of 60 μs in STN DBS is clearly challenged, although future research needs to confirm these encouraging findings.

### Typical Side Effects in STN-DBS

**Figure 3:**
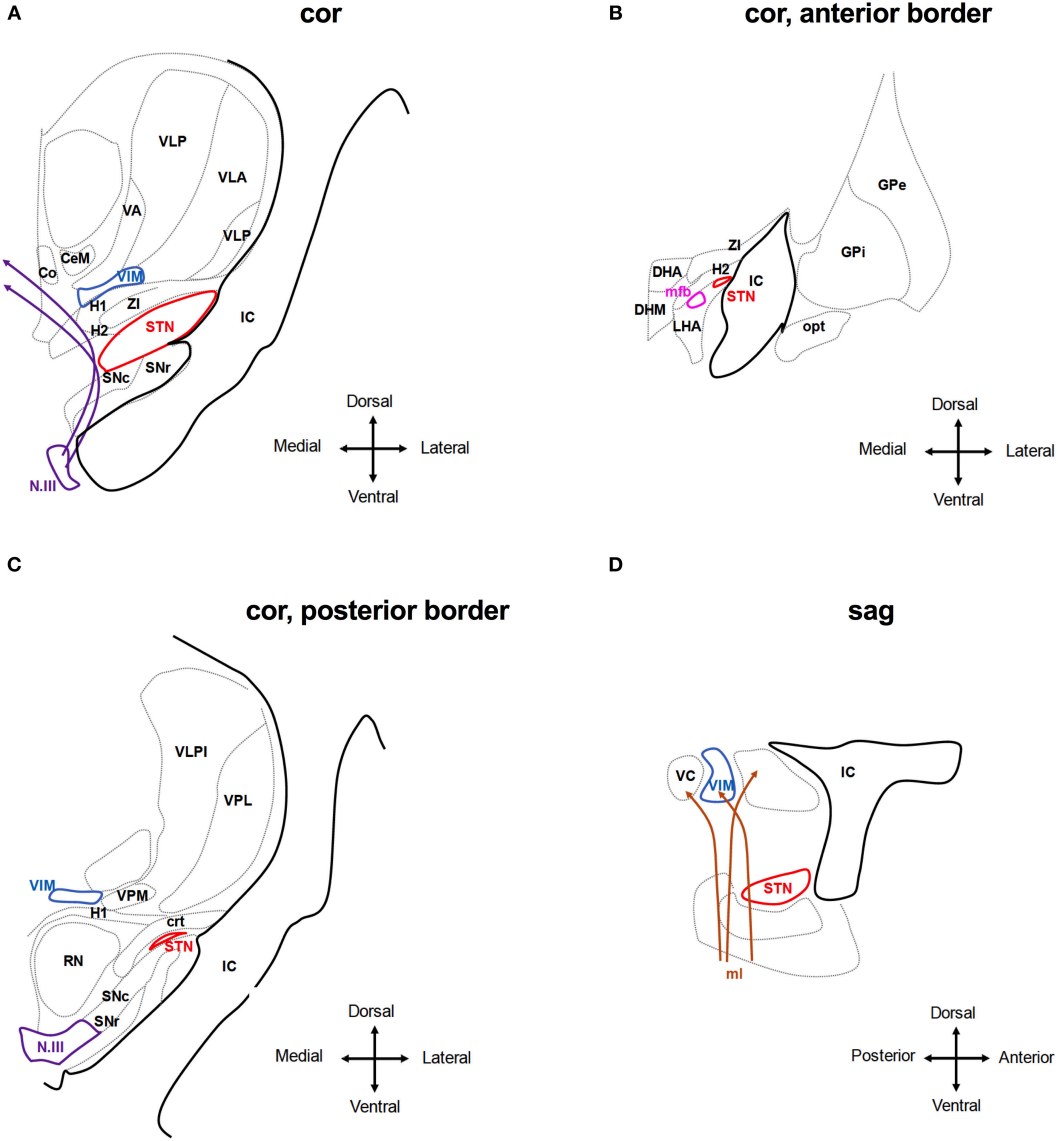
Anatomical relationship of the subthalamic nucleus (STN) and the ventral intermedius nucleus (VIM) to adjacent structures. The schematic shows coronar **(A–C)** and sagittal **(D)** planes through the basal ganglia at the level of the STN and VIM. Co, Commissural nucleus; CeM, central medial thalamic nucleus; VA, ventroanterior thalamic nucleus; VC, ventrocaudal nucleus; VLP, ventrolateral posterior thalamic nucleus; VPM, ventroposterior medial thalamic nucleus; IC, internal capsule; SNr, Substantia nigra pars reticulate; and SNc compacta; H1, H2, H1 and H2 Fields of Forel; ZI, zona incerta; N.III, nucleus of the third cranial nerve; DHA, dorsal hypothalamic area; DHM, dorsomedial hypothalamic nucleus; LHA, lateral hypothalamic area; mfb, medial forebrain bundle; opt, optic tracts; RN, red nucleus; crt, cerebello-rubro-thalamic tract. Stimulating the tissue medial and dorsal to the STN activates the *H1* and *H2* fields of Forel and the ZI and may reach to the medio-dorsal thalamic nuclei incl. the Co, CeM, and VIM. Deflection of the field to more ventral areas will activate the fibers of the N.III and the SN **(A)**. Anterior of the STN, stimulation may activate hypothalamic nuclei and mfb as well as the IC **(B,D)**. At the posterior border of the STN, stimulation may activate the RN and ml, in particular, if the tissue medial of the STN is activated. Stimulation of tissue dorsal of the STN may activate the crt. **(C,D)**. Adjusted from Mai et al. (55).

Most DBS side effects can be understood as a result of current spreading into brain regions adjacent to the target area. The STN is a relatively small, ovoid structure with a close anatomical relationship with other deep brain nuclei and tracts, including the *internal capsule* (lateral, anterior), the *substantia nigra* (ventral), the *red nucleus* (medial), *the fibers of the third cranial nerve* (medioventral), *the thalamic fasciculus*, also termed field H1 of Forel and composed of the ansa lenticularis and the lenticular fasciculus (mediodorsal), the *sensory thalamic nuclei* (dorsal), the *zona incerta (ZI) and cerebello-rubro-thalamic fibers* (medial dorsal, posterior), and the *hypothalamus and medial forebrain bundle* (anterior) (55, 56) (Figure 3). In addition to these anatomical relationships, the STN is subdivided into different territories (motor, oculomotor, associative, and limbic), each with different connections and specific functions (57). Previous studies that have analyzed the anatomical location of the most effective contacts used for chronic stimulation showed varying results: the majority of reports suggest that the most effective contacts to ameliorate PD symptoms segregate to the dorso-lateral, sensorimotor aspect of the STN (58–64), whereas current spread to the limbic and associative sub-segments may cause unwanted affective and cognitive side effects (65–68). Conversely, other studies recommended targeting other areas or even adjacent regions such as the zona incerta (ZI) or the Forel fields H1/H2 (69–76) and one study found no significant association between the position of the active contacts and the clinical effect (77). This heterogeneity may be a consequence of methodological differences among the studies, as different imaging techniques were applied to define the position of the electrodes including ventriculography, CT and MRI (78). In addition, classical studies applied numerical coordinates referenced to the stereotactic space to define the contact position, making the results difficult to interpret without knowing the patient's individual anatomy and because a volume of tissue is represented by a single point. The following adverse effects in STN-DBS can be derived from the function of the adjacent anatomical structures:

*Spastic muscle contractions*: The most frequent adverse effects include (spastic) contractions involving the facial muscles (“facial pulling”), which often affect bilateral upper facial and contralateral lower facial muscles (79, 80) and are a consequence of current spread into the internal capsule (IC) lateral and anterior to the STN (Figures 3A,D). By modeling the electric field caused by STN-DBS, it was found that even small deviations in the electrodeposition within the STN can result in activation of large diameter myelinated IC axons over a volume that spreads outside the borders of the STN (81).

*Uni- or bilateral gaze deviation*: Typical oculomotor side effects are reduced gaze ipsilateral to stimulation, sometimes progressing to contralateral gaze deviation. This resembles conjugate eye deviation during frontal epileptic seizures and is therefore assumed to be caused by activating fibers stemming from the frontal eye field (FEF) which run in the internal capsule in three bundles: a dorsal trans-thalamic trajectory, an intermediate bundle crossing the subthalamic region, and a ventral bundle in the medial portion of the cerebral peduncle, which projects, among other structures, to the subthalamic nucleus (82). Analyzing 22 electrode locations which intraoperatively could elicit conjugate eye deviations, these positions were found to lie within the lateral anterosuperior border of the STN (Figures 3A,D). This resulted in the recommendation to place the lead or deflect the field to a more medial, posterior, and inferior position (83). In a single case, this phenomenon was elicited with the STN contacts which provided the best clinical efficiency and could be compensated by bilateral STN stimulation (84). These eye movements consisted of several saccades and were accompanied by turning the head. Thus, contra-versive and conjugate eye deviation cannot be generally taken as evidence for electrode misplacement. Conversely, activating the fibers of the third nerve (N.III) that run inferomedial to the STN and within the red nucleus (RN) below the STN may result in unilateral gaze deviation and diplopia (Figures 3A,C). Tamma and co-authors claim that stimulation of oculomotor fibers causes adduction or reduced abduction or elevation of the superior eyelid in the ipsilateral *eye* (85). Also, in another report, unilateral eye deviations were frequently seen during intraoperative test stimulation when the electrode was medial, posterior, and ventral to the final target (46). However, this far medial position makes unwanted stimulation of these fibers an extremely rare instant. In experimental stimulation of the third nerve in macaques, only small adduction of the eye was seen but prompt miosis, as expected from physiology (86). Eyelid opening apraxia has also been observed (51), although this symptom may be present as part of PD itself, and is occasionally relieved by stimulation but also can be induced by stimulation above the clinically efficient threshold (87). Mydriasis is rather frequently seen during intraoperative test stimulation and post-operative adjustment along with ipsilateral perspiration. These are quickly adapting symptoms and are not considered as evidence for a misplaced electrode. The central sympathetic tract runs medial to the red nucleus anteriorly to the aqueduct and is therefore not involved, but sympathetic fibers within the zona incerta (ZI) (88) or within the STN (Figures 3A,B) are assumed to be stimulated when mydriasis occurs.

*Autonomic side effects*: Nausea and excessive sweating are likely a consequence of medial and anterior current spread, presumably corresponding to tissue activation in the hypothalamus and red nucleus (85, 89) (Figures 3B,C). Approximately half of all STN-DBS cases experience dizziness, a sense of heavy- or lightheadedness, or malaise (51).

*Paresthesia*: Contralateral paresthesias may be due to stimulation of the medial lemniscus which conveys somatosensory information from the joints and skin and lies ventroposterior to the STN (Figure 3D). With the usual frontal entry of the lead the lowermost contacts may thus encroach on this structure (89). Mostly, paresthesias are transient but when they persist, a more dorsal contact may be chosen, if clinically effective.

*Speech impairment:* The impairment of speech frequently occurs during the initial programming and long-term follow-up of STN DBS (37, 90) but can be ameliorated through proper programming (91). Dysarthria occurs in about 25% of STN-DBS cases and may be caused by current spread into the internal capsule (strained or spastic speech) or otherwise into the pallidal and cerebello-thalamic fiber tracts (crt) medial and dorsal of the STN (92–94) (Figures 3A,C,D). It is therefore important to distinguish the different causes of DBS-induced dysarthria to be able to adjust stimulation contacts and parameters. In addition, stimulation of the STN itself may account for speech impairment. In particular, medial left-sided stimulation in right-handed patients had a negative effect on prosody, articulation, and overall intelligibility (95–97). Accordingly, higher left STN voltage is associated with deterioration of speech (98). Similarly, other reports demonstrated a strong correlation between high voltages in the left STN and speech impairment (99–101).

One report suggested high stimulation frequency to increase the risk of speech impairment (102). Another report suggested high-frequency stimulation to have a negative effect on speech-related velopharyngeal control (103).

*Dyskinesia*: STN-DBS may induce dyskinesia, such as choreiform, ballistic, or dystonic movements reminiscent of levodopa-induced dyskinesia (52). Dyskinesias occurring during the initial post-operative programming period are thought to indicate a good outcome and the contact inducing dyskinesia is usually the most effective in ameliorating motor symptoms (52, 104–106). Rare dystonic effects in STN-DBS included dystonia of head and neck muscles with stridor and dysphagia (107, 108).

*Gait impairment and postural instability:* Overall, L-Dopa responsive axial symptoms are also more likely to improve with STN-DBS and indeed, various studies reported gait improvement with STN-DBS (109–115), in particular in terms of gait velocity and amplitude of arm and leg swing. On the other hand, long-term follow-up studies (116, 117) have consistently shown that axial symptoms including gait may worsen over time in contrast to the sustained improvement of cardinal motor signs, suggesting a differential effect of DBS on the distal and axial neural control circuits (118–120). Indeed, increasing the stimulation amplitude can worsen gait and increase freezing episodes similar to no stimulation as discussed further in detail in section Specific Programming Strategies to Counteract Side Effects in STN-DBS. However, the cause of gait impairment in DBS is most likely multifactorial (121) and, apart from stimulation-induced worsening through the current spread, disease progression, medication reduction, and cognitive decline may contribute. Postural instability is the least likely to respond to DBS and STN-DBS appears to be more detrimental to postural stability as compared to GPi-DBS (122, 123). Although there is no evidence to support a certain programming strategy to avoid worsening of postural stability, a recent study suggested that limiting current spread to the non-motor territories of the STN would liberate cognitive resources that could be used to maintaining a steady posture (124, 125) and to improve postural stability (126). Because certain studies suggested that trunk ataxia to be a consequence of activating the red nucleus, directing the current to more lateral areas might be also helpful.

*Acute neuropsychiatric side effects*: STN-DBS may cause acute neuropsychiatric alterations in addition to preexisting psychiatric comorbidities that can decompensate during or after surgery (106). Neuropsychiatric signs can be observed in individual subjects during initial programming and may include apathy (112, 127), mirthful laughter (66) as well as acute mania (68, 128) and acute depression (129–131).

*Depression*: In a case described by Bejjani et al., depression occurred while all contacts were screened in the post-operative setting. When contact the most ventral (Figures 3B,C) was activated, depression set in after 5 s. of stimulation with 2.4 V. This contact was not efficient in relieving PD symptoms and was shown to be located within the substantia nigra. Stimulation of more dorsal contacts provided relief from PD motor signs without causing depression. In addition, apathy and depression may be due to a “hypodopaminergic” state as a consequence of a quick or radical reduction in dopaminergic medication (132). Recognizing depression is highly relevant since these symptoms have an even bigger impact on the live quality of DBS patients than motor function (133, 134).

*Mania*: Manic episodes due to STN stimulation are assumed to be a consequence of stimulating the medial and ventral aspects of the STN (135, 136). Therefore, the use of more dorsal contacts is recommended in these cases. In addition, stimulating tributary fibers from the STN to the median forebrain bundle may contribute to these symptoms (65).

*Impulse Control Disorders (ICD)*: The relationship between DBS and ICD is complex and in part controversial (137). In general, bilateral STN-DBS was found to either ameliorate or worsen decision-making or to have no effect (138–140). STN-DBS is associated with the risk of binge eating (141, 142) and punding behavior (143). Moreover, STN-DBS may induce hypersexuality, hypomania (144, 145), or compulsive gambling (146). These effects are most likely associated with using the most ventral contacts (147–150) and are assumed to be caused by stimulating the ventromedial, limbic area of the STN (66, 149, 151) as well as the SNr (128) and the medial forebrain bundle (65) (Figures 3A,C). One therapeutic option may, therefore, be to avoid current spread into STN-related limbic circuits by deflecting the electrical field to more dorsal and lateral parts. However, ICD may also resolve or improve after surgery (152, 153) and STN-DBS might in fact be considered to treat ICD in PD (152, 153). Long-term follow-up of patients with STN-DBS showed pre-surgery ICD was abolished in most patients once L-DOPA or dopamine agonist doses were reduced (141) as was the dopamine dysregulation syndrome (154). In these studies, the *de-novo* onset of ICD was rare and transient with the exception of compulsive eating (141). Similar to motor symptoms, the individual patient outcomes in regard to ICD depend on several factors, including target selection, electrode location, programming settings, appropriate medical management, age, and perhaps genotype (155) and is thus difficult to predict.

*Cognitive side effects*: The effects of STN-DBS on cognition remain controversial. A reduced verbal fluency is well-described (156), but has been observed with and without stimulation and thus has been attributed to penetrating the caudate nucleus during surgery (157, 158). On the other hand, Morishit et al. and Isler et al. found no significant difference in cognitive decline between caudate-penetrated and caudate-spared groups. In addition, executive dysfunction and altered short term memory have been observed (159, 160). These effects are also considered to be a consequence of stimulating the ventral and medial aspect of the STN (160, 161). However, well-controlled studies did not find detrimental effects of STN-DBS on global cognitive function (162, 163). The etiology is therefore likely multifactorial and due to the surgical lesion of the frontal lobe and caudate nucleus and diseases progression (164).

### Specific Programming Strategies to Counteract Side Effects in STN-DBS

Some adverse effects may be transient in nature and will disappear despite continuing stimulation (165). For instance, dyskinesia is a typical side effect of STN-DBS in PD but increasing the amplitude in minute steps and waiting for the dyskinetic symptoms to disappear after each incremental step might ultimately allow for an increase in amplitude required for symptom control despite transient dyskinesia (105). Moreover, it may be sufficient in some instances to adjust stimulation parameters in order to achieve a more symmetrical or asymmetrical DBS effect. For example, if gait disturbances are prominent in STN-DBS, reducing the stimulation amplitude on the side contralateral to the best motor response resulted in increased stride length, reduction of gait variability, and a reduction in freezing episodes (166). On the other hand, asymmetric stimulation may be helpful in ameliorating the emotional side effects of STN-DBS, that are thought to be lateralized (167). The latter study demonstrated emotional auditory stimuli to induce activity in the ventral non-oscillatory region of the right STN but not in the left ventral STN or in the dorsal regions of either the right or left STN. These results suggest that DBS of the right ventral STN may be associated with beneficial or adverse emotional effects observed STN-DBS. The authors suggest that the stimulation parameters in the right STN should be modified to counteract psychiatric side effects. This hypothesis is tempting but needs further confirmation from clinical studies. When permanent side effects occur, either the stimulating contact or the stimulation parameters may be changed or, as the last option, the electrode may be repositioned. The first step is to check the electrode position in case this is not done routinely after surgery or if post-surgical images are not available. The second step is to reduce the current of the activated contact(s) and/or choose another contact for stimulation. For example, choosing a more dorsal contact is recommended when persistent paresthesias occur as well as in psychiatric symptoms (see above). Alternative electrode configurations can be achieved by combining single contacts to a compound cathode (double or triple monopolar) or by setting another lead contact as an anode (bipolar). The latter allows the volume of tissue activated (VTA) to be restricted at the expense of higher energy consumption (3), although one should be aware that the extent of the computed VTA varies substantially with the material properties of the surrounding brain tissue (168–171). Alternatively, interleaving stimulation (Medtronic®) may be applied. Interleaving stimulation (ILS) consists of rapid and alternate activation of two electrode contacts with two distinct amplitudes and pulse widths but with the same frequency up to a maximum of 125 Hz and a delay of 4 ms between two stimuli. In general, ILS may be applied either to limit stimulation-induced adverse effects or else, to stimulate different brain regions with individualized settings in order to alleviate specific symptoms (47). For example, ILS was successfully applied for freezing of gait (additional stimulation of substantia nigra) (101) as well as tremor (additional stimulation of zona incerta) (172). However, with the exception of case reports and small case series (172–177), there are no larger prospective trials that have investigated the clinical effect of ILS. In accord with previous reports, a recent study from Kern et al. demonstrated improvement with ILS for adverse effect management predominately for the treatment of dyskinesia and improvement of PD motor symptoms with ILS (178), whereas ILS was less effective in ET and dystonia. Of note, a contact was added into the rostral zona incerta (ZI) (Figure 3A) in the majority of dyskinetic patients, thus suggesting a particular role of the ZI and the surrounding pallido-thalamic fibers for improving dyskinesia and a potential ILS target in STN-DBS. These alternative targets are under active investigation for treating dyskinesias (174, 179, 180), although sound evidence for using these structures is still lacking. A drawback of ILS is that battery drainage is likely increased with ILS as 2 independent programming settings are applied (181).

#### Short Pulse Width Stimulation (SPWS)

Decreasing the standard pulse width, which is currently only possible with Boston Scientific® or Abbot® devices, represents an alternative strategy to counteract unwanted side effects in STN-DBS (53, 182). For example, Reich et al. investigated pulse widths below 60 μs at a frequency of 130 Hz and found that compared to (standard) 60 μs stimulation, the therapeutic window increased by a mean of 182% with a PW of 30 μs, and decreased by 46% with a PW of 120 μs (183). Although the stimulation amplitude required for rigidity control increased with reducing pulse widths from a mean of 1.6 mA at 60 μs to 2.9 mA at 30 μs, the TEED required for the clinical effect of rigidity control decreased. This is thought to be mediated by more selective action of stimulation on the fiber tracts that are responsible for symptom relief while the neighboring thick and myelinated corticospinal and corticobulbar fibers are thought to be less affected by short pulse width stimulation (184–186).

#### Low-Frequency Stimulation (LFS)

If gait and balance issues such as freezing of gait (FOG) or other axial symptoms predominate, LFS (60–80 Hz) may be a good treatment strategy for PD patients with STN-DBS. FOG is a gait disorder featured by recurrent transient gait retardation and interruption that occurs in PD, PD-plus syndromes and vascular parkinsonism. Most FOG episodes are related to the OFF state in PD, but severe cases begin to suffer from ON state FOG (ON-FOG). FOG increases the risk of falls for PD patients and has a large impact on the motor function and daily life of the patients. HFS-DBS of the STN can alleviate FOG in some patients, particularly if FOG is related to medication OFF state (187–189). On the other hand, HFS-DBS may induce FOG in PD (190, 191). Pharmacological treatment options for FOG include L-DOPA (192), methylphenidate and amantadine (193, 194). Alternatively, the stimulator may be switched to LFS. LFS (60–80 Hz), compared to HFS (130 Hz), has been shown to have beneficial effects on improving FOG and other axial symptoms, such as speech and swallowing function, in PD patients with bilateral STN-DBS in some studies (190, 191, 195–198) or selected patients (199), but not in others (200–203). Some found short-term but not long- term beneficial effect (204), while others found both short-term and long-term benefits after 6 weeks, 8 months and even 10 months study periods (190, 195, 197). It is not well-understood, which factors account for the different responses of FOG and other axial symptoms to LFS. Possible factors include the presence or absence of pre-existing FOG, the frequency used (60 vs. 80 Hz), the maintenance of the TEED [TEED = (voltage^2^ × pulse width × frequency)/impedance)] with frequency adjustment and the location of the active contacts (ventral vs. dorsal). In most studies, adjusting for TEED appeared to be less relevant than the frequency (205). This in line with the finding that neuronal responses relative to frequency are highly non-linear as demonstrated by Huang et al. (206). In summary, it is currently unclear, which patients benefit most from LFS vs. HFS, but likely applies to patients that have pre-existing FOG at HFS-DBS on exam (190, 195–197). In some studies, switching from a high to low frequency (<100 Hz) stimulation also ameliorated speech intelligibility (207) and acoustic parameters such as hypophonia (196). On the other hand, tremor control has been observed to be worse with lower frequencies (190, 197, 204).

#### Alternative Electrode Targets for Axial Symptoms and Gait Disorders

If there is a beneficial effect of LFS on gait, it may be caused at least in part, by affecting neurons that project to the pedunculo-pontine nucleus (PPN) as unilateral or bilateral LFS of this structure directly and in combination with stimulation of additional target structures has been shown to improve FOG (99, 187, 208–213). The PPN has reciprocal cholinergic connections with the STN, its degeneration may be crucial in the pathophysiology of gait and balance deterioration in PD (214, 215) and stimulation of the PPN may improve axial symptoms in PD (216, 217). The PPN may be stimulated by leads in this region alone or in conjunction with the STN, the SNr, or the GPi (99, 211, 212). Interestingly, the optimal contact positions for LFS were more ventrally located in the STN than optimal contacts for 130 Hz-stimulation (198). More recently, there has been interest in the stimulation of the SNr, which is located ventrally and medially to the STN (218). One study found that among PD patients treated with STN-DBS at 130 Hz via the most distal contact of the quadripolar electrode resulted in an improvement of gait and posture (100). Subsequently, another group of researchers used interleaving to stimulate both the STN and the SNr (101) and found that FOG was significantly improved with combined STN/SNr stimulation, although other axial symptoms on UPDRS did not significantly differ. At the same time, stimulating the SN also comprises the risk of worsening akinesia and of inducing depressive symptoms. In summary, the combined stimulation of PPN plus STN, PPN plus GPi, or STN plus SNr, may be useful for the treatment of FOG in PD patients. The optimal combination of nuclei to be stimulated and the stimulation parameters need to be determined by future clinical trials. In addition to its effect on gait and balance, LFS may reduce stimulation-induced dyskinesia (219, 220). This may be particularly relevant for dorsal-projecting contacts in or close to the ZI above the STN, that have been reported to have an anti-dyskinetic effect with different stimulator settings (178, 221, 222).

### Optimal Initiation Time for Programming and Adjusting Pharmacotherapy in STN-DBS

#### General Considerations on Post-Operative Care

The time point to initiate DBS after STN implantation surgery varies between institutions. Early programming (within the first days after surgery) satisfies the patient's wish for a timely treatment but may be hindered by a improvement in PD symptoms due to the lesion caused by the electrode (stun effect) which may last up to 2 weeks (223, 224) or even longer: the mean medication “ON” time improved 3 months after STN electrode implantation even in the absence of electrical stimulation (115), thus demonstrating an improvement with surgery alone. At which time point DBS is initiated after surgery thus depends on the procedures established in each institution. In any way, the initial programming should be performed after an overnight washout of dopaminergic drugs so that the effect of DBS can be assessed without the interference of medications (37). Adjusting anti-parkinsonian drugs typically occur after initial programming of STN-DBS. There is no specific evidence on how and when to adjust medication after STN-DBS is programmed. The insertional effect and the effect of the electrical stimulation synergize to ameliorate PD symptoms, thus requiring a reduction of the pre-operative LED to avoid dyskinesia. In addition, there may be significant placebo or nocebo effects subsequent to electrode implantation. Stopping dopaminergic medication altogether is not recommended, as this may induce a hypodopaminergic state including apathy and depression. Importantly, these symptoms may develop even weeks after the cessation of dopaminergic drugs (225–227). In particular, in patients that suffer from impulse control disorder, cutting dopamine agonists is advisable (152). Otherwise, L-Dopa should be reduced first (228, 229). Finally, reducing L-Dopa might unmask preexisting Restless Legs Syndrome that would have to be considered for treatment.

#### Constant Voltage vs. Constant Current Stimulation

In addition to the micro-lesion effect, the fluctuation of impedances may bias the determination of the therapeutic window in the early post-operative period (230) which might become more relevant hen using constant-voltage stimulation (CVS) where the current delivered is inversely proportional to the electrode impedance. Conversely, current-constant stimulation (CCS) may offer more stable stimulation, in particular when programming soon after surgery (231, 232). Apart from possibly affecting the outcome in an individual patient, using CCS instead of CVS might allow for an improved generalization of outcome between subjects such that knowledge gained from one set of subjects can be generalized to others. Because the total current delivered current depends on both voltage and impedance, and since voltage is held constant with CVS, potential variations in current over time will be mainly a consequence of impedance fluctuations. Data from examining non-human primates using a small version of the human DBS lead supported this hypothesis (233) as the electrode impedance progressively increased over 7 days post-implantation, resulting in a reduction of current delivered. Benabid et al. reported impedance changes in patients with VIM stimulation for ET. These authors observed an increase in impedance of 33% (on average) over 3 months following the implantation of DBS leads. Thereafter the impedance stabilized (234). Sillay et al. measured impedances in 63 DBS patients with PD, essential tremor, and dystonia at various time intervals following DBS surgery (235). All measurements were performed at >25 days post-operatively, and in the absence of changes in the stimulation parameters between time points. On average, the authors found no significant intra-patient or intra-electrode impedance changes. However, over half had a small increase in impedance over time, and 40% had a small decrease in impedance, with the largest change observed being 23% in a single subject. Hemm et al. described similar results in patients with dystonia (236) observing that impedance values changed only slightly over time within a single patient but that there were differences between patients and between active and non-active DBS contacts. However, Cheung et al. analyzed a large database of impedance measurements from 94 subjects, ranging from 6 months to 5 years after implantation. They found that a significant amount of impedance variability could be expected in chronically implanted DBS electrodes, with a range spanning from 18 to over 600 Ω (237). Studies that compared CCS and CVS did not show any significant differences in non-motor outcomes, including cognition, mood, and quality of life in a double-blind crossover trial (238). A retrospective analysis of 19 patients with PD and dystonic syndromes switched from CVS to CCS reported no change in measured clinical outcomes and therapy satisfaction at 6 months (115, 239), whereas a more recent study found better outcomes with CCS (240). Taken together, the relevance of changes in the electrode impedance and, as a result, the total electric charge transferred, is uncertain and the specific consequences of using CCS vs. VCS stimulation are not yet clear (231, 241, 242) and currently, there is no clear evidence to support an early or late post-operative initiation of DBS.

### VIM-DBS in Essential Tremor

#### Specific Programming Strategies in VIM-DBS

Compared to STN-DBS, the evidence for adjusting neurostimulation parameters in VIM-DBS is limited. In case of ET, kinetic tremor, the principal target of stimulation adjustments, the limb can be assessed with the finger-to-nose or finger-to-finger maneuver or by asking the patient to draw a spiral, drink water from a cup or pour water from a glass into another one. In addition, postural tremor can be assessed with the arms outstretched or elbows bent (wing-beating position). In general, the programming strategies outlined above can be applied for VIM-DBS. Using a pulse width of 60 μs and a frequency of 130 Hz, the current intensity is usually increased progressively until tremor stops or until side effects are encountered. If the tremor is not optimally controlled at 3.5 volts, pulse width and then the frequency of the stimulation may be increased (243). Studies evaluating the effect of different stimulation parameters in ET showed that tremor responds best to increase the amplitude and is further improved by 25% with longer pulse widths (90–120 μs). The frequency-response curve shows an inverse linear relationship between tremor magnitude and frequency between 45 and 100 Hz and a plateau above 130 Hz, although an additional but variable effect between 130 and 200 Hz has been documented (2) (244–247). Similar to what has been demonstrated for STN-DBS, reducing the pulse width has been shown to widen the therapeutic window in ET (248) where the minimum pulse width for suppression of tremor was shown to be significantly different to that for induction of ataxia, with values of 27 and 52 μs, respectively (249). Comparing directional stimulation with segmented electrodes to conventional ring stimulation, Rebelo et al. found an increased therapeutic window and reduced current with stimulation in the best direction compared to the best omnidirectional stimulation alternative (44) (Figure 1). Likewise, alternative targets directly adjacent to the VIM have been described for ET. For instance, the caudal ZI has been examined as a target for patients with tremor suggesting that ZI stimulation may even exceed tremor control through stimulation of the VIM (250–253). These findings are consistent with results from diffusion tensor imaging data suggesting that the best tremor control is obtained with stimulation of the cerebello-thalamic afferents, which are embedded in the ZI (249).

#### Typical Side Effects in VIM-DBS

The VIM nucleus of the thalamus is located close to the STN in the vicinity of the *internal capsule* (lateral), the *centromedian and parafascicular nucleus* of the thalamus and the *commissural nucleus* (medial), the *zona incerta (ZI) and H1/H2 field of Forel* (ventral), *the ventroanterior (VA), the ventrolateral anterior (VLA) and posterior (VLP) nuclei of the thalamus* (dorsal), and the *ventromedial thalamic nucleus (VM)* (anterior, posterior) (55) (Figure 3). Common side effects include the following:

*Paresthesia* is the most common short term side effect because the electrical field reaches into the thalamic sensory nuclei dorsal to the VIM (Figure 3A). It can be transient, lasting from a few seconds to minutes, or permanent, and only resolving with reducing stimulation (2, 234, 254).

*Speech impairment:* Dysarthria is a significant complaint in more than half of ET patients with bilateral VIM-DBS (255), although dysarthria is common in ET even in the absence of DBS. This is relevant because clinicians often choose suboptimal stimulation parameters to avoid stimulation-induced side effects, more frequently seen in patients with bilateral VIM-DBS (255, 256). Speech impairment appears to occur more frequently with higher stimulation amplitudes and with more ventral stimulation contacts. As with STN-DBS, dysarthria may be caused by interference with the cerebello-thalamic or with motor fibers of the internal capsule (Figures 3C,D) located laterally to the VIM causing spastic dysarthria (257) and appropriate contact adjustment may be beneficial.

*Gait ataxia*: Another common complaint in patients with VIM-DBS is balance issues with an unsteady gait. As with speech disturbances, current spread into dentato-thalamic afferents lateral and ventral the VIM (Figure 3C) may be the cause of such gait and limb ataxia (258–260), although gait and limb ataxia can be a sign of ET itself, commonly referred to as ET-Plus (261). Switching off DBS even for several days can help to distinguish between the two, but rebound tremor needs to be considered.

*Loss of Stimulation Benefit*: In ET, the energy required for tremor suppression and the number of active contacts typically increase as the disease progresses and this effect is more common in ET as compared to other tremor types (262–265). Indeed, some studies showed the initial improvement in activities of daily living evident at 1 year after the DBS implantation to be lost in the long run except the ability to eat (266). The loss of long-term benefit in ET has been attributed to DBS tolerance, natural disease progression, and other factors including brain atrophy (234, 266–271). Possible strategies to avoid the adaptation of neuronal networks in ET include switching the stimulation off at night (255), inverting the electrode configuration in patients using bipolar settings or on-demand stimulation.

### GPi-DBS in Generalized and Segmental Dystonia

#### Specific Programming Strategies in GPi-DBS

GPi-DBS has been applied worldwide as a surgical treatment alternative for medical refractory segmental or generalized dystonia. Although GPi-DBS seems to be more effective for isolated than non-isolated dystonia (272), there is no evidence that non-isolated dystonia needs a different programming approach (273–275). The role of specific stimulation parameters on dystonic symptoms is probably even less established than with VIM-DBS for ET. This is likely a consequence of the heterogeneity of symptoms. In addition, and unlike in STN- and VIM-DBS, where the effect is observed within seconds to minutes, the effect of GPi-DBS on dystonia may not occur for hours, days, or in some cases even months (Figure 2B). For instance, Krauss et al. noted that phasic dystonic movements were often relieved within minutes of stimulation onset, whereas improvement in tonic posturing took several months to fully manifest (276). When adjusting neurostimulation in dystonia, phasic dystonic movements, such as dystonic neck movements, are therefore best suited for evaluation because tonic dystonic components usually need more time to improve (277). This may be in part be due to musculoskeletal abnormalities caused by long-standing dystonic posture. Accordingly, most GPi-DBS patients fail to show a clear insertional effect (277). In accord, tonic dystonic symptoms may take a lot longer to reappear upon cessation of GPi-DBS than phasic one (278–281) (Figure 2B). In some cases, discontinuing GPi-DBS may result in a clinical rebound effect with acutely severe symptoms (282, 283). The principal programming algorithm follows the same recommendations as with PD or ET with some modifications (3). For instance, a high frequency of 185 Hz has been proposed to be effective in GPi-DBS (284). There is a debate on the selection of the contact for chronic stimulation as there is a poor correlation between benefit and stimulation in different regions of the GPi. Cheung et al. recently identified a small area located squarely in the middle of the GPi as a potential specific therapeutic target for DBS for dystonia (285), whereas recent evidence from our own group suggests that most efficient DBS electrodes displayed a close anatomic proximity to the pallidothalamic tracts (ansa and fasciculus lenticularis) between the GPi and the pyramidal tract (286). Thus, stimulation is most commonly initiated in the ventral region of the GPi above the optic tract (contacts 0 and 1) (287) with a short pulse width (60–120 μs), high frequency (130–185 Hz) and amplitude just prior to eliciting adverse effects (284, 288, 289). Due to the anatomical location of the target, delayed side effects are less likely to occur than with STN- or VIM-DBS, thus favoring a top-down approach and starting the stimulation with the highest tolerated voltage. The use of high- vs. low-frequency stimulation in dystonia has shown mixed results. Alterman et al. suggested that the use of 60 Hz stimulation can be beneficial in some patients (290), whereas another group preferred high-frequency stimulation (289). Moro et al. concluded that high-amplitude and high-frequency stimulation predict better outcome in cervical dystonia (291). Various pulse widths have been recommended in GPi-DBS. Coubes et al. recommend the use of 450 μs (292). However, another study comparing 60, 120, and 450 μs did not show any significant differences between the three groups (293).

#### Typical Side Effects in GPi-DBS

**Figure 4:**
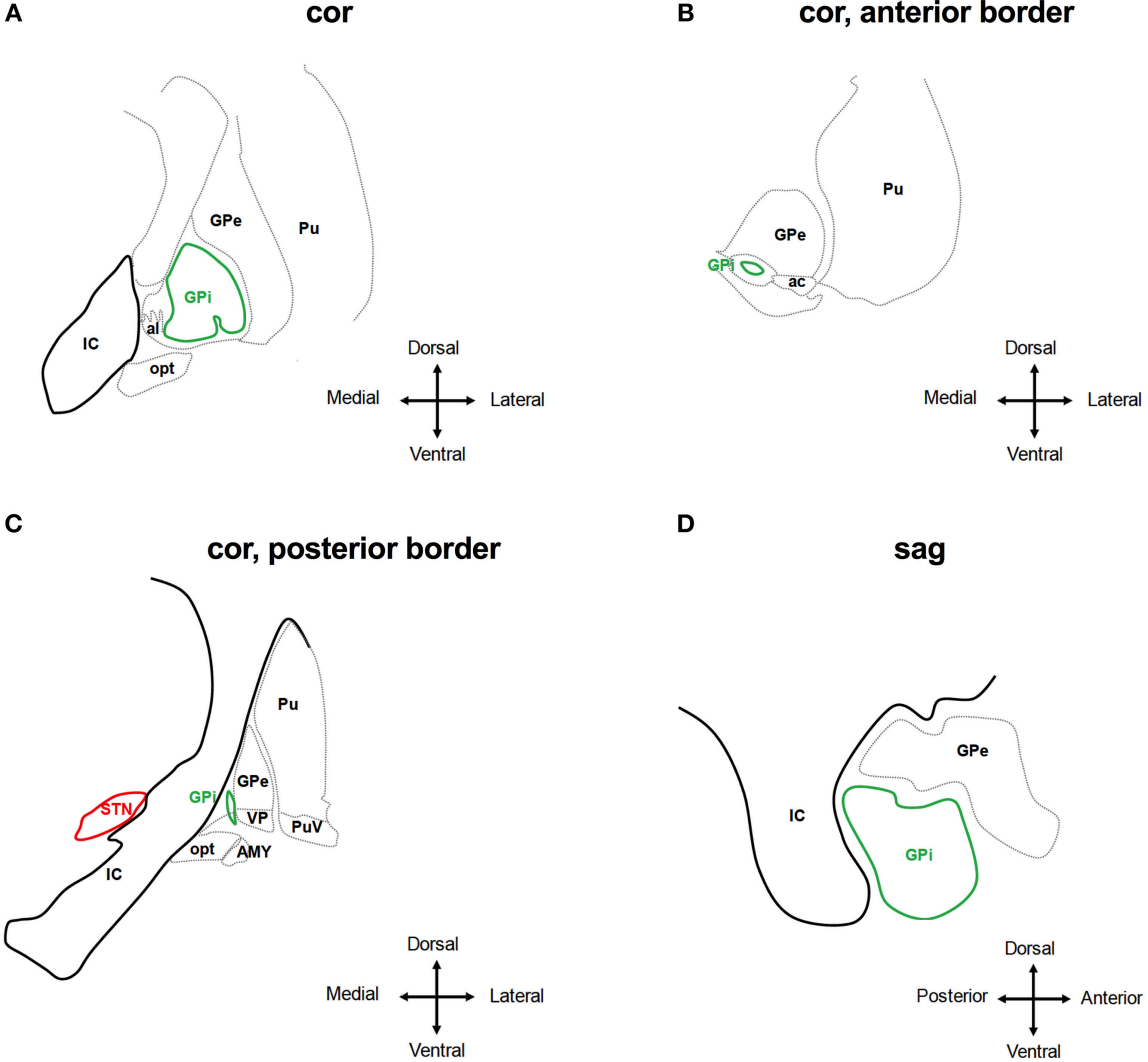
Anatomical relationship of the globus pallidus internus (GPi) to adjacent structures. The schematic shows coronar **(A–C)** and sagittal **(D)** planes through the basal ganglia at the level of the GPi. IC, internal capsule; GPe, globus pallidus externus; al, ansa lenticularis; Pu, putamen; opt, optic tract; AMY, amygdala; VP, ventral pallidum; PuV, ventral putamen; STN, subthalamic nucleus. Deflection of stimulation to tissue medial of the GPi will activate the IC, which is less likely the case at the anterior border of the GPi **(A,B,D)**. The AMY and opt are activated by stimulating tissue ventral of the GPi **(C)**. Adjusted from Mai et al. (55).

The GPi is surrounded by the *globus pallidus externus and putamen* (anterior, posterior, lateral), the *internal capsule, ZI and MFB* (medial), the *ansa lenticularis* (mediodorsal), the *optical tract* (ventral), the *amygdala* (laterodorsal), the *ventral pallidum* (laterodorsal) (55) (Figure 4). As with STN- and VIM-DBS, side effects in GPi stimulation can result from current spreading into neighboring regions in many cases:

*Hypo-/Bradykinesis:* The occurrence of parkinsonian motor signs, such as micrographia and postural deficits, has been described as a possible adverse effect of GPi-DBS in dystonia (294–297). This may be the result of stimulating distinct regions within the GPi: whereas stimulation of the dorsal part of the GPi improves PD signs and symptoms like hypokinesia and rigidity, stimulation of the postero-ventral part suppresses levodopa-induced hyperkinesias but may lead to a deterioration of hypokinesia and gait (284, 298). As a consequence, stimulation-induced hypokinesia is more frequent with use of the ventral contacts and may be significantly reduced by switching to dorsal contacts. Because the ventral contacts are the most effective at controlling dystonic symptoms, this approach may lead to a worsening of dystonia (294, 299, 300).

*Speech Impairment*: In patients with primary dystonia treated with GPi-DBS, dysarthria is one of the most common stimulation-induced side effects reported in close to 30% in follow-up studies (277, 301). As with STN- or VIMN-DBS, this may be caused by current spreading into the internal capsule medial and posterior to the GPi (Figures 4A–D). In addition, stuttering may occur with GPi stimulation (257, 302), emphasizing the role of the GPi in speech fluency.

*Phosphenes*: These may be caused by current spread into the optic tract that is located ventral of the GPi (Figures 4A,C).

There is no specific evidence for general programming strategies to avoid speech disturbances in GPi-DBS other than the general strategies for avoiding side effects outlined above.

## Conclusion

Programming the IPG is the only modifiable factor once DBS leads have been implanted and thus crucially impacts on the overall treatment success. Although our review does not provide a specific level of evidence for an overall programming strategy, we here summarized appraised strategies on how to adjust stimulation parameters and program settings in different movement disorders. Therefore, we reviewed previous studies that examined the significance of distinct stimulation strategies for ameliorating disease signs. We summarized the well-characterized significance of the stimulation amplitude, frequency and pulse width on clinical symptoms. In addition, we provided an in-depth review of potential side effects in DBS of the STN, VIM, and GPi. Based on these effects, we specifically examined more recent techniques for modulating neuronal elements, such as directional current steering, low-frequency, and short pulse-width stimulation as these strategies were shown to enlarge the therapeutic window and thus allow for a more favorable outcome in different movement disorders. In conjunction with a recommendation for managing pharmacotherapy in PD after initiation of DBS, we thus provide a concise review for STN-, VIM-, and GPi-DBS programming.

## Author Contributions

TK conceived the project, conducted literature research, and wrote the paper. CP, FH, JM, and KB wrote the paper.

### Conflict of Interest Statement

The authors declare that the research was conducted in the absence of any commercial or financial relationships that could be construed as a potential conflict of interest.
